# Network- and enrichment-based inference of phenotypes and targets from large-scale disease maps

**DOI:** 10.1038/s41540-022-00222-z

**Published:** 2022-04-26

**Authors:** Matti Hoch, Suchi Smita, Konstantin Cesnulevicius, David Lescheid, Myron Schultz, Olaf Wolkenhauer, Shailendra Gupta

**Affiliations:** 1 Department of Systems Biology and Bioinformatics, University of Rostock, 18055 Rostock, Germany; 2 Heel GmbH, 76532 Baden-Baden, Germany; 3 Leibniz-Institute for Food Systems Biology, Technical University of Munich, 85354 Freising, Germany

**Keywords:** Computational biology and bioinformatics, Molecular medicine

## Abstract

Complex diseases are inherently multifaceted, and the associated data are often heterogeneous, making linking interactions across genes, metabolites, RNA, proteins, cellular functions, and clinically relevant phenotypes a high-priority challenge. Disease maps have emerged as knowledge bases that capture molecular interactions, disease-related processes, and disease phenotypes with standardized representations in large-scale molecular interaction maps. Various tools are available for disease map analysis, but an intuitive solution to perform in silico experiments on the maps in a wide range of contexts and analyze high-dimensional data is currently missing. To this end, we introduce a two-dimensional enrichment analysis (2DEA) approach to infer downstream and upstream elements through the statistical association of network topology parameters and fold changes from molecular perturbations. We implemented our approach in a plugin suite for the MINERVA platform, providing an environment where experimental data can be mapped onto a disease map and predict potential regulatory interactions through an intuitive graphical user interface. We show several workflows using this approach and analyze two RNA-seq datasets in the Atlas of Inflammation Resolution (AIR) to identify enriched downstream processes and upstream transcription factors. Our work improves the usability of disease maps and increases their functionality by facilitating multi-omics data integration and exploration.

## Introduction

### Background

Molecular and cell biology has amassed a tremendous amount of information on molecular interactions related to disease development, progression, and treatment. Clinical scientists and biomedical researchers have access to any chosen disease phenotype, process, or molecule through databases built on scientific literature and experimental data. However, searching publications and databases for molecules of interest and identifying regulatory mechanisms and potential drug targets is—in most practical cases—a long-term research project rather than a quick task.

### The disease map approach

**Fig. 1:**
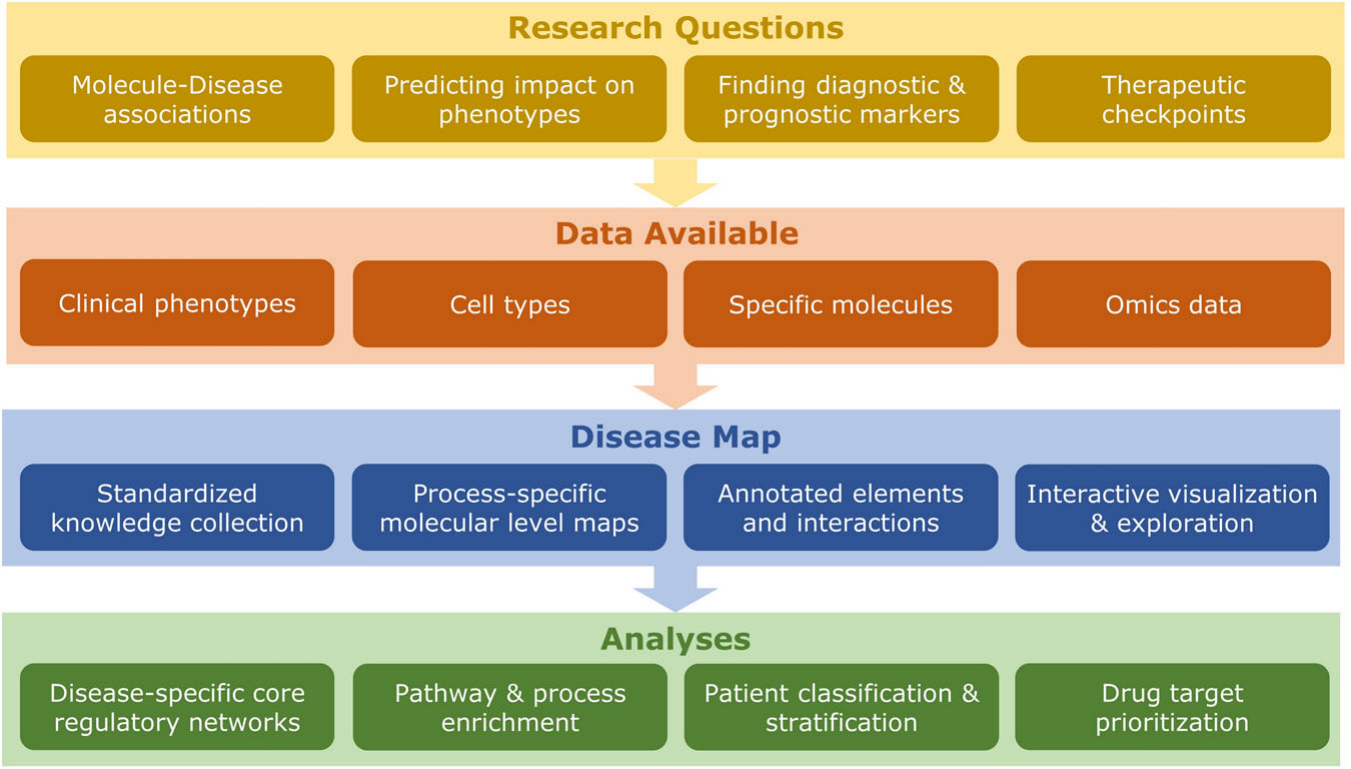
Overview of the disease map approach to address disease-specific research questions, as implemented within the suite of tools developed in the present work. Disease maps are context-specific. The starting point is thus the selection of the targeted phenotype, molecules, or networks of interest. The goal may be the search for diagnostic markers or therapeutic checkpoints. The information that is used to curate the disease map, comes from a variety of sources, covering information about clinical phenotypes, cell types, specific molecules of interest, and experimental data. Information about molecular interactions is encoded using standardized formats. The analyses can then be conducted with the suite of tools presented in this paper.

Disease maps are developed to support the disease-oriented exploration of state-of-the-art knowledge. Community-built disease maps are comprehensive and accessible resources that collect validated knowledge about a disease, its molecules, phenotypes, and processes^1,2^. Encoding this knowledge in a standardized format enables established analytical tools to extract information from the complex interactions or perform in silico experiments on integrated experimental data (Fig. 1). Examples of published disease maps include the Parkinson’s Disease Map^3^, the Rheumatoid Arthritis Map^4^, the AsthmaMap^5^, the Atherosclerosis Map^6^, and the COVID-19 Disease Map^7^.

**Fig. 2:**
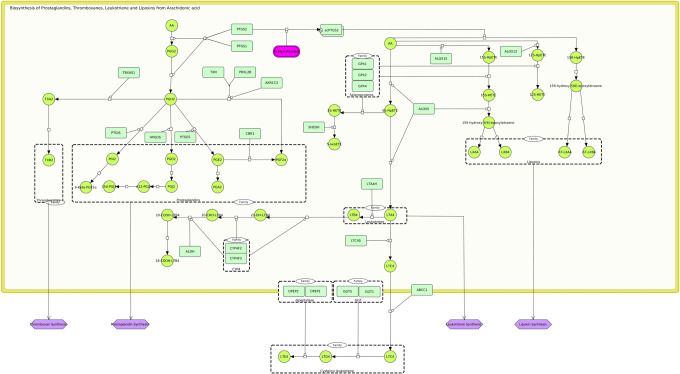
SBGN representation of the “biosynthesis of PIM and SPM from AA” in the “atlas of inflammation resolution” (AIR). Molecular interactions represented in the SBML process description format are involved in the regulation of various phenotypes (purple) such as “thromboxane synthesis” or “prostaglandin synthesis.” The advantage of such standardized representations is that they can be analyzed using bioinformatics and systems biology approaches, including graph-theoretical analyses of the topological structure of large networks, statistical analyses, logical and mechanistic modeling, and simulation.

Systems biology standards encode contextual and visual information, such as Systems Biology Markup Language (SBML), Systems Biology Graphical Notation (SBGN), or CellDesigner-SBML, which can organize molecular interactions into diagrams and layers^8–10^. Usually, disease maps consist of multiple, functionally organized diagrams, so-called submaps, that describe the molecular interactions regulating related biological processes or clinically observable signs and symptoms, represented as SBGN phenotype elements. Elements of these submaps can be linked to public databases using stable identifiers and organized into different layers that aid in the visualization and exploration of disease maps. Figure 2 gives an example of a submap from the “Atlas of Inflammation Resolution” (AIR)^11^.

The curation of submaps is a manual process that aggregates experimentally validated evidence from the literature and provides a rich annotation of interactions with links to various databases. In the AIR, the submaps are programmatically extended with protein-protein interactions (PPI) and regulatory information, including transcription factors (TF), microRNA (miRNA), or long non-coding RNA (lncRNA) interactions. The curation of the AIR has been described previously^11^. The entirety of molecular interactions, the “bottom layer” of the disease map, combining information from submaps and regulatory interactions, we refer to as the molecular interaction map (MIM). The MIM encodes information about molecules and their interactions in pathways, networks, and their relationship to disease phenotypes. Even for a narrowly defined context, most disease maps will include large numbers of interactions. To make disease maps publicly accessible and interactive to the community, MINERVA was developed as a web-based platform for curating and interactively visualizing disease maps that support community-driven projects^12^. Because it enables automated annotation with multiple databases and extensive exploration tools, MINERVA hosts many currently published disease maps, including the AIR. Additionally, MINERVA allows data mapping and coloring of corresponding elements in the submaps, and thereby, the intuitive exploration of experimental measurements such as changes in gene expression, metabolite concentrations, or genetic mutations.

### Research gap

The development of tools for disease map analyses has many challenges, given their complex nature and the wide range of proposed applications. These challenges include, for example, applying them on large-scale networks and having minimal restrictions to include various biological data types. From a computational perspective, such tools should enforce data security guidelines, be made easily accessible, and be implemented into an intuitive user interface. Because of these challenges, disease maps analysis has been limited and typically requires implementing external tools into the workflow. Consequently, data must be exported, transformed, and again imported, which requires knowledge of programming languages and limits the usability of disease maps for non-bioinformaticians.

Increasing the analytic power of methodologies usually comes with decreasing applicability. Established approaches such as ODE or Boolean models—although providing more detailed simulations—require many efforts to prepare the desired subpart of the network and can be very computationally extensive. Hence, one of the most used approaches is enrichment analysis, which is computationally effective and has many applications in commonly used tools, including DAVID^13^, ClueGO^14^, or Enrichr^15^. Its simplest form, the overrepresentation analysis (ORA), evaluates the statistical overrepresentation of a user-supplied list of input elements in predefined sets of elements^16^. Typically, the input list consists of differentially expressed genes (DEGs) from RNA-seq or microarray experiments, while the predefined sets contain genes linked to phenotypes. In this way, ORA can analyze whether, for example, genes related to a particular disease are overrepresented in the analyzed data. The Enrichr web platform provides a simple user interface for ORA, harnessing many public databases for generating gene sets, including disease databases such as the human phenotype ontology (HPO)^17^ or pathway resources such as KEGG^18,19^ and WikiPathways^20^. However, ORA is limited in its interpretability. It provides a statistical evaluation of overrepresentation, but no information about (i) the type of regulation (up- or down-regulation), (ii) the relationships between genes and the enriched entity, (iii) the range of fold changes, or (iv) the importance of each gene (its weighting) in the set. The “Gene Set Enrichment Analysis” (GSEA) extends the ORA approach by ranking the input genes by their fold change values and analyzing whether their up- or downregulated genes are overrepresented. Several commonly used analysis tools, such as GeneTrail^21^, have integrated the GSEA approach. Still, GSEA does not evaluate the relationship between the genes and the enriched element. Numerous enrichment approaches have addressed these limitations to broaden their scope for specific purposes. They distinguish between up- and down-regulated interactions (BD-Func) or integrate network topology information into their algorithms (network-weighted GSEA)^22–24^. The “Reverse Causal Reasoning approach” (further referred to as RCRA) integrates network information of upstream elements and statistically analyzes whether their regulatory directions correspond to the fold change directions^25^. However, RCRA does not include fold change values of genes in the list and only considers direct upstream regulations, restricting applications of the approach. In 2014, QIAGEN published the “Ingenuity Pathway Analysis” (IPA) software that provides a range of network-based solutions to infer knowledge from molecular data^26^. Like RCRA, IPA considers only directions of gene expression regulations. However, IPA additionally analyses downstream effects, includes multiple steps in the network, and implements a more sophisticated statistical analysis. IPA is similar to the disease maps approach because it visualizes molecular pathways and provides data integration and analysis tools. Still, it has been designed for commercial use, limiting its use in academic community-driven projects.

In summary, none of the current approaches incorporate sufficient information into their algorithms. Either information on the input list (fold change values and direction) or on the relationship between the inputs and the enriched elements is missing. One of the reasons for this could be the lack of such information, since most databases store gene sets without further information about the relationship between entities. Second, the inclusion of continuous values (e.g., fold changes) that are not normally distributed complicates statistical analysis. GSEA, for example, solves this problem by using running sum statistics with gene set permutation to analyze enrichment along with ranked fold changes (one-dimensional). Even if all these issues are resolved, using these approaches for disease maps remains a challenge. Users would need to generate gene sets from the map manually and import them into the enrichment tools together with their data. For some enrichment approaches, this would require an additional coding step, as they may not have a ready-to-use implementation. These limitations compromise data integration and force users to re-export the enrichment results to the disease map for visualization. Such a workflow is contrary to the principles of disease maps, which envision intuitive and straightforward web-based implementations. Therefore, there is a need for tools that enable in silico experimentation, data integration, and data visualization directly on disease maps through intuitive and simple user interface elements.

### Outcomes

We developed a two-dimensional, network-based enrichment analysis (2DEA) approach that, through the combination of topology and data-integration methods, facilitates deriving information from complex, large-scale networks such as disease maps. Since MINERVA supports customized plugins that can interact with the displayed submaps, it provides an excellent framework for community-driven and application-focused projects^27^. By integrating our approaches in a multifunctional, interactive MINERVA plugin suite within the AIR, we help users answer their research questions on the map itself and visualize results in colored overlays of map elements. To demonstrate applications of the tools, we derived regulated phenotypes from a bulk RNA-seq dataset of a murine colitis model^28^. Additionally, we applied the upstream enrichment to an RNA-seq dataset of IFNα-stimulated B-cells and identified well-known transcription factors activated by IFNα as targets^29^. Both case studies demonstrated the successful identification of regulated processes and known key targets.

### Terminology

Theoretically, any input type can be applied for enrichment approaches, not only DEGs. In this work, we extend the definition of the input list to a list of arbitrary elements with quantitative (level-based) or qualitative (activity-based) changes, which we refer to as differentially changed elements (DCEs). DCEs are elements characterized by a significant *log*2 fold change value (FC), either derived from transcriptomics, proteomics, or metabolomics experiments (data-dependent DCEs) or simply assumed by the user (data-independent, in silico simulated DCEs). DCEs can also be phenotypes, referring to increased (positive value) or decreased (negative value) activities of measurable biological processes or clinical features. Additionally, we redefined the to-be enriched element as any element that is either regulated by the DCEs (downstream enrichment) or itself regulates the DCEs (upstream enrichment). The enrichment is positive or negative depending on the direction of the DCEs’ fold change and their relationship to the enriched element. From that perspective, upregulation of positively associated elements or downregulation of negatively associated elements has the same net positive effect. Conversely, in the case of negative enrichment, the fold changes and the associations should be oppositely directed.

## Results

### The two-dimensional enrichment analysis (2DEA)

**Fig. 3:**
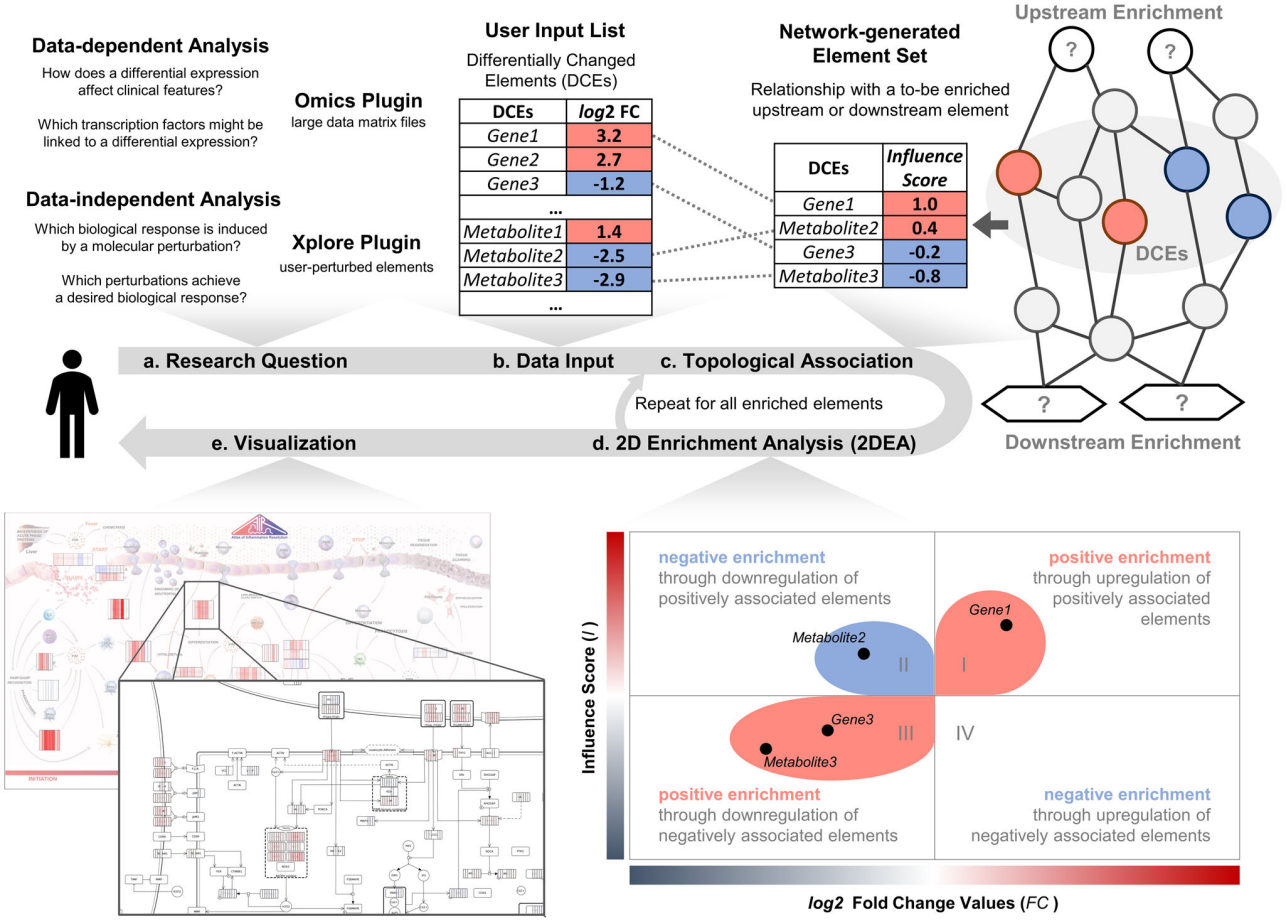
Summary of the two-dimensional enrichment analysis (2DEA) approach and its implementation as a disease map plugin. **a** We have developed two plugins for the MINERVA platform that allow user interaction and perform in silico perturbation analysis on disease maps. Depending on the research question, perturbed elements come either from large experimental data files (Omics plugin) or from elements on the map individually selected and perturbed by the user (Xplore plugin). **b** In both cases, the inputs can be viewed as a list of differentially changed elements (DCEs) characterized by an FC value. **c** The DCEs are mapped to the molecular interaction map and their topological relationship to (downstream) or from (upstream) the element to be enriched, represented as a numerical value called Influence Score. **d** 2DEA then statistically evaluates whether the combination of FC values and influence scores is overrepresented towards positive enrichment (same direction) or negative enrichment (opposite direction). **e** Enrichment scores, FC values, and influence scores can be presented intuitively to the user as colored overlays on standardized network diagrams and images in MINERVA.

Figure 3 summarizes the 2DEA approach and its implementation as a disease maps analysis tool. The approach is described in detail in the method section. 2DEA distinguishes between up- or downregulation of positively- or negatively associated elements by combining information on quantified input elements (fold changes) with the weighted relationship to the element that will be enriched (influence scores) (Fig. 3b, c). Because both variables are continuous and not normally distributed, the statistical analysis becomes challenging, which we solved by identifying the significance of the variable distribution in the two-dimensional space among randomized input (Fig. 7). Thereby, 2DEA can statistically evaluate whether an enriched up- or downstream element is positively or negatively enriched in the input data (Fig. 3d). Other enrichment approaches usually do not or only partially include this information, as shown in Table 1.

**Table 1:** Comparison of 2DEA with other established enrichment approaches.

Approach	Reference	Downstream	Upstream	Fold change direction	Fold change value	Regulatory direction	Regulatory weighting
ORA^38^	Boyle et al.						
GSEA^16^	Subramanian et al.						
RCRA^25^	Catlett et al.						
IPA^26^	Krämer et al.						
ROMA^39^	Martignetti et al.						
PADOG^40^	Tarca et al.						
Weighted GSEA^24^	Zito et al.						
BD-Func^23^	Warden et al.						
2DEA							

^a^Although the respective approach has not been described as applicable for up- or downstream analysis, the enrichment analysis can theoretically be applied for both.

The table highlights whether each algorithm considers information from the input list (fold change direction or value) or information on the relationship between items in the input list and the enriched element (regulatory direction or weighting).

**Fig. 4:**
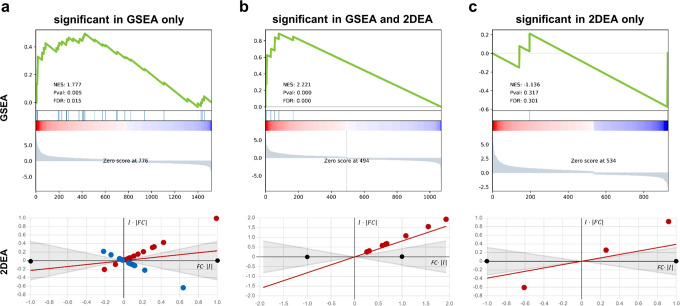
Comparison of graphs of GSEA (top) and 2DEA (bottom) enrichment results using a case study dataset. We used GSEA and 2DEA to identify enriched phenotypes in AIR from an RNA-seq dataset using differentially expressed genes (DEGs; adj. *p* value < 0.05) generated by DESeq2 as an input gene list. Three results were selected that were significantly enriched in GSEA only (**a**), in both approaches (**b**), or in 2DEA only (**c**). In the panels of GSEA, a running sum of enriched scores is generated over the list of DEGs, ordered by their *log*2 fold change (FC) value from upregulated (red, left) to downregulated (blue, right). In 2DEA, normalized FC values (x-axis) are linked to network topology-based influence scores (I, y-axis) to identify the distribution of DEGs in either the direction of positive (red) or negative (blue) enrichment.

**Fig. 5:**
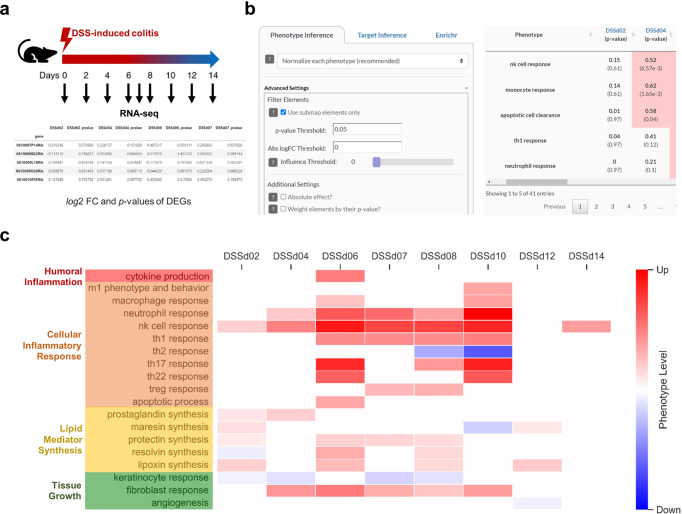
Downstream enrichment analysis with the Omics plugin to identify regulated phenotypes in a murine colitis model. **a** Using DESeq2, differentially expressed genes (DEGs; adj. *p* value < 0.05) were identified from a colon bulk tissue RNA-seq dataset of mice with DSS-induced colitis for eight different time points (Czarnewski et al., 2019). **b** In the Omics plugin, the phenotype inference was performed by filtering the DEGs for elements that occur in submaps of the AIR. Results are presented in an interactive table, showing predicted levels and *p* values and creating phenotype regulator plots for each entry. **c** Heatmap of significantly regulated phenotypes in each sample, normalized for each phenotype separately.

To show how differences in integrated information affect the results of enrichment approaches and their interpretability, we compared 2DEA with GSEA in a case study. We analyzed a bulk tissue RNA-seq dataset from a murine colitis model (Fig. 5a)^28^. As the DCE input list for both enrichment approaches, we identified significant differentially expressed genes (DEGs; adj. *p* value < 0.05) in all eight samples using the DESeq2 R package. For every sample, we applied 2DEA as well as GSEA to enrich all 42 phenotypes in the AIR. The gene sets associated with each phenotype were the same for both approaches, i.e., all elements within the AIR MIM that have an influence score on the enriched phenotype that is nonzero. We then selected three enrichment results, one significant only in GSEA, one significant in both approaches, and one significant only in 2DEA. Figure 4 shows the output graphs of both approaches for each of the selected results. The creation of the 2DEA graph is described in detail in the Methods section (Fig. 7). For an explanation of the GSEA panel, we refer to the 2005 paper by Subramanian et al.^16^. In Fig. 4a, the enrichment by GSEA, but not by 2DEA, is significant. Although upregulated DEGs are overrepresented (left side of GSEA panel and right side of 2DEA panel), these DEGs have ambiguous effects (similarly distributed positive and negative influence values). GSEA cannot assess the relationship between DEGs and enrichment phenotype and thus identifies a significant overrepresentation of upregulated DEGs. In Fig. 4b, DEGs are also upregulated but all with positive influence values, so both 2DEA and GSEA identify significant enrichment. In Fig. 4c, GSEA predicts false negatives when upregulated and downregulated elements are equally represented. However, upregulation of DEGs with positive influences and downregulation of DEGs with negative influences can be considered the same result and vice versa. The 2DEA shows its strength by accounting for these correlations and allows such cases to be predicted as significant.

The use of influence scores in 2DEA allows enrichment to be weighted based on the importance of the DCEs. This gives 2DEA an advantage over other network-based enrichment approaches such as BD-Func or IPA that integrate only non-weighted regulatory directions. For example, when upregulating an element that is positively associated with a phenotype, BD-Func does not distinguish whether there is a close relationship or not. If the element is one of the strongest and closest regulators of the phenotype, it is likely to have biological relevance of interest for the user. 2DEA improves the statistical evaluation and, thus, facilitates their interpretation of molecular data by integrating causal relationships from molecular networks.

### A plugin suite for disease map knowledge inference

We present a suite of MINERVA plugins, initially developed for the Atlas Inflammation Resolution (AIR) but adaptable for other disease maps as well. The plugins can be accessed directly from the AIR (https://air.elixir-luxembourg.org/) and are thus easily accessible from any web browser (Supplementary Fig. 1). The two central components of the plugin suite are the Xplore and Omics plugin, both of which integrate the 2DEA approach. The plugin suite builds an interface for users to apply molecular perturbation either through manual selection or data integration, perform enrichment analyses, and, finally, intuitively present results in colored overlays (Fig. 3a, e).

The Xplore plugin provides data-independent solutions to explore disease mechanisms in silico. It allows users to detect changes in downstream phenotypes based on perturbed elements or identify common upstream regulators by defining the desired phenotype state. Easy-to-use UI elements and color-based visualization facilitate the use of the tools and the interpretability of their results (Supplementary Fig. 2). Because the user inputs involve few elements and serve the purpose of exploration rather than full-fledged analyses, we reduce customizations of methods and details of results in the Xplore plugin to avoid over-complication. The plugin extends the primary purpose of disease maps, which is: to present knowledge about diseases to the public in a user-friendly form, with tools to perturb molecules or define a biological state by tracking its effects or causes in the system.

We developed the Omics plugin with sophisticated enrichment tools that may provide insights into the biological and molecular environment of large molecular data files supplied by the user. We provide detailed information on the results by graphically displaying the DCEs in each enriched set, intuitively highlighting elements of interest on the submaps, and allowing multiple options for statistical analysis. Users can adjust the parameters of the algorithms or define thresholds for DCEs that fit their data. In addition, we provide an automated optimization function to identify settings with as many filtered DCEs for the highest thresholds as possible. However, we emphasize that interpretations of the results should always happen in the context of experimental settings. A detailed explanation of the algorithms is available in the method section.

### 2DEA infers modulated downstream phenotypes from a murine colitis model

To demonstrate data-dependent inference of phenotypes from the plugins, we analyzed the same dataset as we used for the comparison with GSEA. As input to the Omics plugin, we summarized the results in a tab-delimited.txt file containing the official gene symbol with the respective FC and adjusted *p* values generated by DESeq2. Using the plugin, we then identified significantly regulated phenotypes (*p* value < 0.05 by 2DEA) for each sample. Figure 5C summarizes the results in a heatmap showing significant upregulation of cellular inflammatory and lipid mediator related processes between day 6 and day 10. Our results are congruent with the findings of Czarnewski and colleagues^28^, who predicted increased immune cell invasion and cytokine production between day 6 and day 10 based on gene ontology (GO) enrichment.

### 2DEA infers upstream regulators of IFNα-induced differential expression

**Fig. 6:**
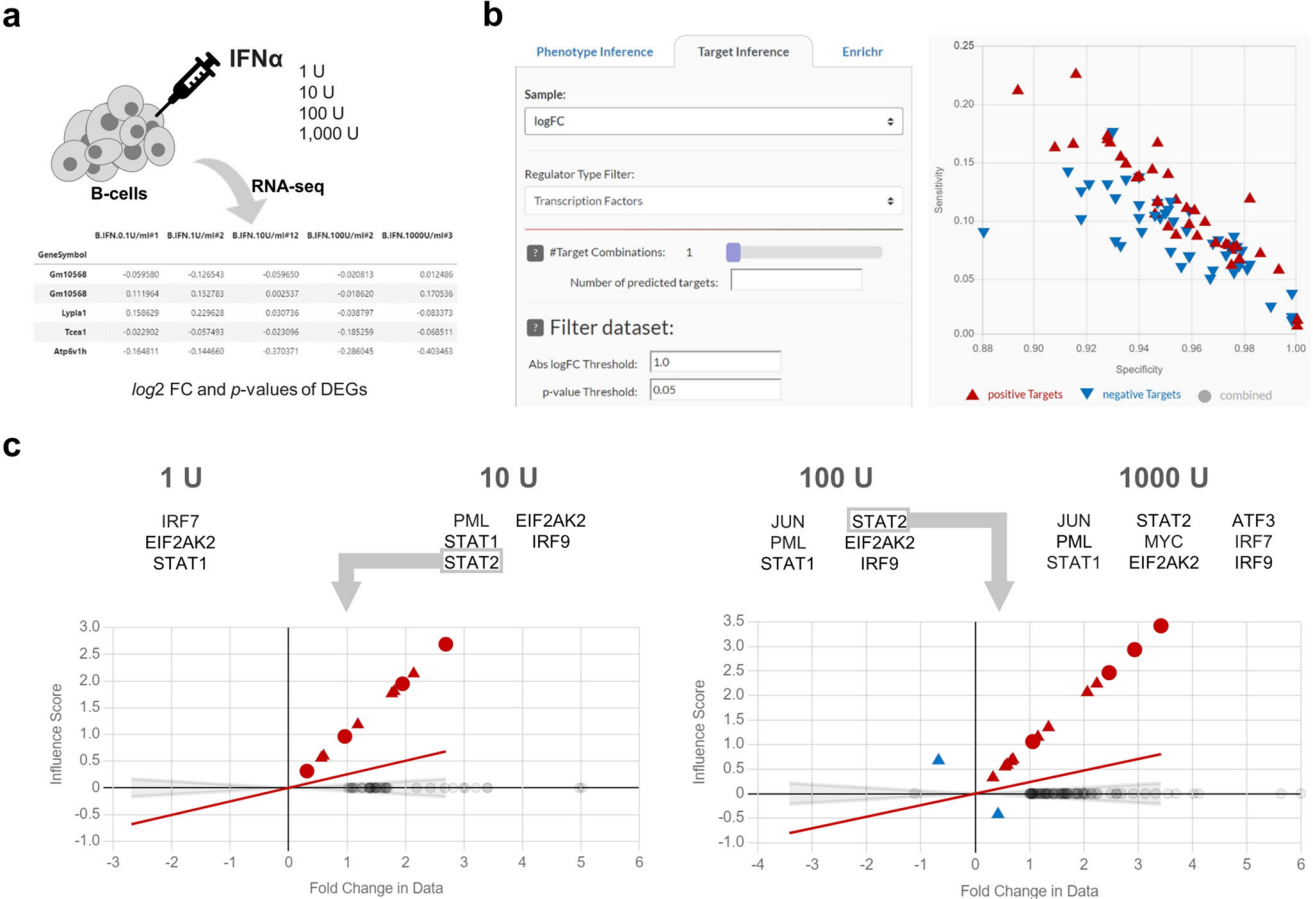
Upstream enrichment analysis with the Omics plugin to identify targets in IFNα induce differential expression. **a** We analyzed the differential expression of IFNα stimulated B-cells for four different concentrations and identified *log*2 fold change (FC) values of differentially expressed genes (DEGs) with an adj. *p* value < 0.05 using GEO2R. **b** We used the target inference tool in the Omics plugin by filtering the DEGs for |FC| >1 and the enriched elements for transcript factors only. The plugin presents the results as an interactive scatter plot showing specificity (x-axis) and sensitivity (y-axis) scores. **c** The highest-ranked, significant, and differentially expressed targets (adj. *p* value < 0.05 by 2DEA) for each sample. Additionally, we show two regulations plots for STAT2 visualizing FC values (x-axis) and transcriptional influence scores (y-axis) of regulated DEGs in the data.

To demonstrate the target prediction through upstream enrichment, we analyzed RNA-seq data from single-cell B-cells stimulated with IFNα in four different concentrations (1 U, 10 U, 100 U, and 1000 U)^29^ (Fig. 6). Significantly differentially expressed genes were loaded into plugins with their adj. *p* values and FC values as generated by GEO2R, summarized in a text file. We performed an upstream enrichment analysis to identify transcription factor targets with significant interactions with the DEGs in the data (see method section). Out of 700 possible TFs in the MIM, we selected TFs with the highest Sensitivity that are also differentially expressed in the experimental dataset. Interestingly, three TFs, namely STAT1, STAT2, and IRF9, reoccurred multiple times among all the samples (Fig. 6c). These TFs are listed as known downstream effectors targets of IFNα in the literature, together forming the Interferon Stimulated Gene Factor 3 (ISGF3) complex^30–33^. In the dataset, 5, 20, 48, and 104 DEGs are defined as TFs in the AIR MIM, respectively. As all these TFs could have been predicted as targets, a reoccurrence of the three ISGF3 TFs by chance would have been very improbable (*p* = 5.61E-9). Figure 6c provides additional insight into calculating results by target-regulation plots illustrating the correlation between FC values of DEGs and their transcriptional influence scores from STAT1 and STAT2, respectively.

## Discussion

Disease maps are increasingly valuable knowledgebases for studying disease mechanisms in silico and providing researchers and clinicians with an interactive platform for data exploration and visualization. We present a two-dimensional enrichment analysis (2DEA) that combines network topology-based relationships between the inputs and the enriched element, called influence scores, with fold change values of input data as weighting factors. The inclusion of both scores allows for more detailed evaluations by assessing the direction and strength of the responses. By integrating the influence scores, we improve the accuracy of the enrichment by giving higher weights to topologically more relevant elements. Additionally, the enriched sets of elements can be generated automatically by filtering influence scores for a defined threshold, thus eliminating the need for manual set curation. That allows for generating weighted enrichment sets from large-scale networks. Even on their own, influence scores are a valuable tool for expanding the information content of disease maps, which provide a visual overview of regulatory processes (Supplementary Fig. 3).

The two-dimensional approach allows for more accurate biological regulations predictions than other enrichment approaches. In molecular biology, many systems or pathways are regulated by the induction of only a few or even a single key enzyme. Conventional enrichment tools cannot detect these cases where individual changes are distributed among different sets. Our approach does not evaluate the probability that a given element list is overrepresented in the set but whether the accumulated influence of these elements relative to their fold change is statistically significant compared to random permutations. In this way, we can detect enrichments with a small number of associated inputs, allowing more accurate predictions. By converting large-scale molecular interaction maps from disease maps into enrichment sets of molecule-phenotype or context-specific molecule-molecule associations, we developed a size-independent network-based solution for disease map analysis. We managed to keep computation times to a minimum so that analyses can be performed on the client-side, avoiding the need to upload or store data precluding any data security issues. The approach is highly customizable in that the algorithm for network-based influence score calculations can be adapted for various disease map types without updating the user interface or enrichment part. This customizability improves enrichment capabilities for different data types, e.g., catalytic influence scores for metabolomics data and transcriptional influence scores for transcriptomics data.

We provide an intuitive solution enabling web-based perturbation experiments and data analysis directly on disease maps with the methodology presented here. We successfully addressed many challenges in developing disease map analytic tools, intending to make our method intuitively usable for any interested researcher. Influence scores can be precalculated and stored on the server, enabling fast analyses with large datasets. Plugins require no data upload and can even be performed offline because they are executed as JavaScript on the web browser, and computation times are minimized. Systems biology approaches should help scientists understand their data and point them to potentially important aspects rather than simply displaying computational results or rankings. The plugin suite focuses on making computations transparent. By incorporating graphical visualization of the DCEs and their weights in the enrichment sets, the plugins provide as much information as possible, helping users interpret the results.

## Methods

### Network preparation

The enrichment analysis is based on a molecular interaction graph *G*, which consists of a set of elements (vertices *V*(*G*)) and their connecting interactions (edges *E*(*G*)). Because elements in molecular networks, especially in disease maps, are usually extensively annotated, we assume that the biological type of each element (protein, metabolite, phenotype, …), as well as interaction (catalysis, transcriptional regulation, positive or negative influence, …) is known. In the reduced activity flow format, the interactions encode whether two elements are linked by (de)activation, up- or downregulation, defined as a collection of triples $E \subset \left( {s \times r \times t} \right)$ consisting of a source element $s \in V$, a relation $r \in \{ - 1,1\}$, and a target element$t \in V$. A path *P* in the MIM of the length $L \in {\Bbb N}$ can be written as the sequence $\left( {u_1\mathop { \to }\limits^{r_1} u_2\mathop { \to }\limits^{r_2} \ldots \mathop { \to }\limits^{r_L} u_{L + 1}} \right)$ with $\left( {u_i,r_i,u_{i + 1}} \right) \in E$. The type $T \in \{ - 1,1\}$ of any *P* is defined as $(r_1 \cdot r_2 \cdot \ldots \cdot r_L)$. The shortest path *SP* between two elements $(u,v) \in V$ is defined as an existing path *P*_*u,v*_ between *u* and *v* where *L*(*p*_*u,v*_) is minimized. *SP*_*u,v*_ is considered *consistent* if there is no alternative *P*_*u,v*_ with the same length but opposite type. For run-time identification of interaction paths in the plugins, we implemented a breadth-first search algorithm. The algorithm calculated *L*(*SP*), *T*(*SP*) for all $(u,v) \in V$ and, for all $(u_s,v_s) \in V$ originating from submaps, the elements along $P_{u_s,v_s}$.

### Influence scores as weighting factors

We used the shortest path information to express the relationship between each pair of elements in the network as a numerical value called influence score. Influence scores depend on the context and origin of the data. The phenotypic influence represents an element’s directed, topological weighting in the curated pathways regulating a phenotype. Transcriptional influence describes the effect of a MIM element on the transcription of a particular gene in transcriptomics data analyses. Correspondingly, the catalytic influence describes the impact of an element on the synthesis of a metabolite in metabolomics data analysis. We provide a detailed explanation of the calculation of each score in the method section. The scores are normalized between −1 and 1, where −1 represents a hypothesized strong negative effect, 0 represents no effect, and 1 represents a strong positive effect from one MIM element to another. The calculation of an influence score *I* between two elements $(u,v) \in V$ in the MIM is based on their connecting paths *P*_*u,v*_. However, the routing of the path depends on the context of the analysis. For example, analyzing transcription data, the shortest path leads through transcription factors of *v*. Or when analyzing metabolomics data, the path goes through enzymes in synthesis pathways of *v*. We differentiate between three different types of influence scores through context-specific paths between *u* and *v*:

1. Transcriptional influence (*I*_*T*_) of *u* on a gene *v* is based on the minimal distance of *u* to *v*’s transcription factors (TF_*v*_) in the MIM (Eq. 1)). If $u \in {{{\mathrm{TF}}}}_v$, its influence is equal to the type of interaction between *u* and *v*, i.e., 1 for gene induction or −1 for gene suppression. If $u \,\notin\, {{{\mathrm{TF}}}}_v$, its influence is calculated by aggregating the transcriptional influence of each $k \,\in\, {{{\mathrm{TF}}}}_v$ on *v* multiplied by the interaction path type of *u* on *k* and divided by their distance as a power of two with $\left| {I_{T_{uv}}} \right| \not > 1$.

1 $$I_{T_{u,v}} = \left\{ \begin{array}{ll} T\left( {SP_{u,v}} \right),&{{{\mathrm{if}}}}\;u \in {{{\mathrm{TF}}}}_v\\ \mathop {\sum}\limits_{k \in {{{\mathrm{TF}}}}_v\atop {L\left( {SP_{uk}} \right) \le 2}} \left( {I_{T_{k,v}} \cdot \frac{{T\left( {SP_{u,k}} \right)}}{{2^{L\left( {SP_{u,k}} \right)}}}} \right),&{{{\mathrm{otherwise}}}} \end{array} \right.$$

2. Catalytic influence (*I*_*C*_) of *u* on a metabolite *v* is based on the minimal distance of *u* to *v*’s synthesizing enzymes (*E*_*v*_) in the MIM (Eq. 2)). *E*_*v*_ also includes upstream catalytic enzymes and enzymes that consume *v*. If $u \in E_v$, its influence is equal to the type of interaction between *u* and *v*, i.e., 1 for synthesis or −1 for consumption. If $u \notin E_v$, its influence is calculated by aggregating the catalytic influence of each $k \in E_v$ on *v* multiplied by the interaction path type of *u* on *k* and divided by their distance as a power of two with $\left| {I_{C_{u,v}}} \right| \not > 1$.

2 $$I_{C_{u,v}} = \left\{ \begin{array}{ll} T\left( {SP_{u,v}} \right),&{{{\mathrm{if}}}}\; u \in E_v \\ \mathop{\sum}\limits_{{k \in E_v}\atop{L\left( {SP_{u,k}} \right) \le 2} } \left( {I_{C_{k,v}} \cdot \frac{{T\left( {SP_{u,k}} \right)}}{{2^{L\left( {SP_{u,k}} \right)}}}} \right),&{{{\mathrm{otherwise}}}} \end{array} \right.$$

3. Phenotype influence (*I*_P_) of *u* on a phenotype *v* is based on the topological inclusion of *u* in paths to *v* (Eq. 3)). $V_s \subset V$ is the set of elements originating from submaps that contain *v*. If $u \in V_s$, its influence is calculated based on the percentage of elements and paths connected with *u*. *N*_*P*_ is the number of all paths to *v* and $N_{P_u} \subset N_P$ are paths that go through *u*. *N*_*V*_ is the number of elements connected to *v* and $N_{V_u} \subset N_V$ the number of elements on the path from *u* to *v*. If $u \notin V_s$, its influence is calculated by aggregating the phenotype influence of each $k \in V_s$ on *v* multiplied by the interaction path type of *u* on *k* and divided by their distance as a power of two with$\left| {I_{P_{u,v}}} \right| \not >\, max\left\{ {\left| {I_{P_{k,v}}} \right|\mid k \in V_s} \right\}$. Finally, influence scores for all phenotypes are normalized by dividing by their maximum absolute value, thereby taking values between -1 and 1.

3 $$I_{P_{u,v}} = \left\{ \begin{array}{ll} T\left( {SP_{u,v}} \right) \cdot \left( {\frac{{N_{{{{\mathrm{P}}}}_u}}}{{N_{{{\mathrm{P}}}}}} + \frac{{N_{V_u}}}{{N_V}}} \right),&{{{\mathrm{if}}}}\;u \in V_s \\ \mathop {\sum}\limits_{{{k \in G_v}}\atop{{L\left( {SP_{u,k}} \right) \,\le\, 2}}} \left( {I_{P_{k,v}} \cdot \frac{{T\left( {SP_{u,k}} \right)}}{{2^{L\left( {SP_{u,k}} \right)}}}} \right),&{{{\mathrm{otherwise}}}} \end{array} \right.$$

### Downstream enrichment

In order to enrich downstream elements, fold changes in DCEs are assumed to be the source or hypothetical cause, and the goal is to identify their effects on other elements in the MIM. This analysis is of particular interest to predict impacts on phenotypes, which we consider the enriched element in the following. Thus, the weighting factors are the influence scores of the DCEs on the phenotype. By aggregating the FC and the influence score values, we obtain a rough estimate of the change in phenotype levels across samples. Because the phenotype level is not an empirical measure, its value is not comparable with other phenotypes. Nevertheless, it provides clues about how the biological process or clinical trait may be regulated across samples. For each phenotype *v* we calculated the estimated change in activity (= level) by aggregating the phenotype influence scores of all regulating elements and their FC value in the given sample (Eq. 4)). Because the phenotype level is based on DCE aggregation, its value depends on the number of elements considered for the analysis. Therefore, we normalize each phenotype by dividing it by its absolute maximum level across all samples.

4 $$\begin{array}{*{20}{c}} {{{{\mathrm{Level}}}}_v = \mathop {\sum }\limits_{u \in {{{\mathrm{DCEs}}}}} (I_{u,v} \cdot {{{\mathrm{FC}}}}_u)} \end{array}$$

Additionally, we provide information on the saturation of the phenotype in the sample, calculated as the percentage of regulators that are DCEs, weighted by their influence score (Eq. 5)).

5 $${{{\mathrm{Saturation}}}}_v = \frac{{\mathop {\sum}\nolimits_{u \in {{{\mathrm{DCEs}}}}} {\left( {\left| {I_{P_{u,v}}} \right|} \right)} }}{{\mathop {\sum}\nolimits_{u \in V} {\left( {\left| {I_{P_{u,v}}} \right|} \right)} }}$$

**Fig. 7:**
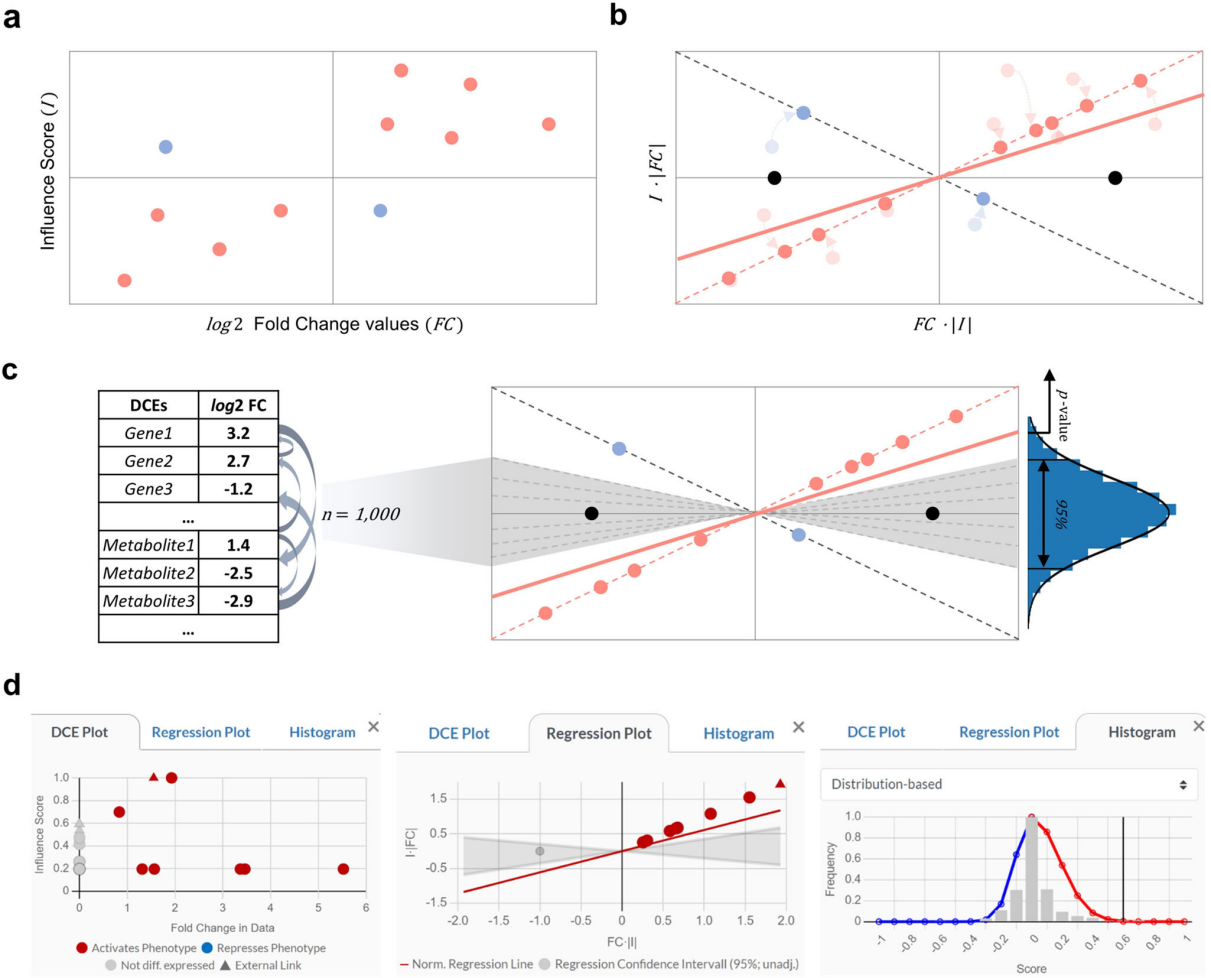
Visual representation of the enrichment score (ES) calculation. **a** Fold change values of elements in the input list and their influence scores are plotted on a graph. **b** All points are shifted on the diagonals with slopes of 1 and −1 (dotted lines), respectively, to normalize their distribution. ES is defined as the regression line’s slope through the origin (red line). Two baseline points (black) are added as a counterweight, forcing the regression towards the x-Axis, making the ES dependent on the number of elements, and ensuring normal distribution. **c** Recalculating ES for randomized input lists (dotted lines) identifies its statistical significance, thus creating a reference null distribution around the x-axis. **d** Screenshots of the AIR plugins user interface that show how statistical features are interactively presented for each result.

For statistical evaluation, we calculate an enrichment score (*ES*, Eq. 6)) that represents the distribution in the *I-FC* plot (Fig. 7a).

6 $$\begin{array}{*{20}{c}} {{{{\mathrm{ES}}}}_v = \frac{{\mathop {\sum }\nolimits_{u \in {{{\mathrm{DCEs}}}}} (|I_{u,v} \cdot {{{\mathrm{FC}}}}_u| \cdot I_{u,v} \cdot {{{\mathrm{FC}}}}_u)}}{{k + \mathop {\sum }\nolimits_{u \in {{{\mathrm{DCEs}}}}} (I_{u,v}^2 \cdot {{{\mathrm{FC}}}}_u^2)}}} \end{array}$$

ES can be thought of as a regression line through the origin in a plot of normalized DCEs versus baseline points. The normalization step projects all points onto the diagonals by fitting them to $({{{\mathrm{FC}}}} \cdot |I|)$ on the x-axis and to $(|{{{\mathrm{FC}}}}| \cdot I)$ on the y-axis, limiting *ES* to a value between −1 and 1 (Fig. 7b). The baseline points are added on the x-axis as a counterweight to force *ES* toward zero and make it depend on the total number of points. The baseline points are represented as *k* in Eq. 6 and provide an individual statistical fit for each DCE set (see upstream analysis below). For the downstream enrichment, *k* = 2 by default, which corresponds to the two points (1,0) and (−1,0) (black dots in Fig. 7b). To identify the statistical significance of the enrichment, we calculated randomized enrichment scores ${\mathrm{ES}}_R = \{ {\mathrm{ES}}_1,{\mathrm{ES}}_2,...,{\mathrm{ES}}_n\}$ and levels ${\mathrm{Level}}_R = \{ {\mathrm{Level}}_1,{\mathrm{Level}}_2,...,{\mathrm{Level}}_n\}$ for *n* random DCE lists (*n* = 1000 by default). The sets are generated for each sample in the supplied data, with an equal number and values of the filtered significant log2 FC values as the original DCEs, randomized among all MIM elements of the same type (e.g., genes or metabolites). Some enrichment studies suggest using term label perturbation instead of gene list permutation to avoid scattering the complex co-expression relationships in the data and thus produce more biologically accurate null distributions^16^. However, because the topological relationships in the network define the weighting factors, even a permutation of term labels would not be an entirely realistic distribution. Therefore, we opted for a permutation of DCEs, which is much less computationally expensive because the number of samples in most cases will be less than the number of enriched elements. Because the influence scores and FC values are not evenly distributed between positive and negative values, it is possible that the normal distribution is different for positive and negative ES values. Therefore, we determine a separate half-distribution for both directions using Gaussian fitting. Then, from the standard deviation σ and mean µ of the identified distribution, the *z*-score for the original ES or Level is calculated (Eq. 7)):

7 $$\begin{array}{*{20}{c}} {z - {\mathrm{score}} = \frac{{{{{\mathrm{ES}}}} - {{{\mathrm{\mu }}}}\left( {{\mathrm{ES}}_R} \right)}}{{{\upsigma }}\left( {{\mathrm{ES}}_R} \right)}}\,{or}\;z - {\mathrm{score}} = \frac{{{{{\mathrm{Level}}}} - {{{\mathrm{\mu }}}}\left( {{\mathrm{Level}}_R} \right)}}{{{\upsigma}\left( {{\mathrm{Level}}_R} \right)}} \end{array}$$

From the *z*-score, the two-sided *p* value is calculated using an iterative approximation based on the Taylor expansion of the distribution’s integral (the code is available at https://air.bio.informatik.uni-rostock.de/plugins). Because the calculation time increases for higher *z*-score values, we set a cutoff to *z*-score = 14, thereby achieving a maximum accuracy for the *p* value of ≈1.56e-44. The *p* value represents the probability of achieving the same or absolutely higher ES or Level than in the original DCE list by random. Finally, we adjust for multiple testing among all enriched elements in each sample using the false discovery rate (FDR)-correction by Benjamini–Hochberg to generate adjusted *p* values^34^. To avoid bias^35^, any user-specific filtering of the enriched elements, in both downstream and upstream enrichment, is performed only after FDR correction.

In the results box, users can choose to display the *p* values from the distribution of enrichment scores, from the distribution of the levels, the highest value from both, or the lowest value from both. For ES statistics, we provide an additional option to automatically adjust the *k* value to the highest FC value in each random set to reduce false negatives in cases where the permuted FC values are higher than the FC values of the original sets, i.e., to prevent nonphysiological FC values from biasing the results. However, as a result of this adjustment, sets with DCEs that have per se high FC values lose statistical power.

### Upstream enrichment

In the upstream enrichment, the fold changes in DCEs are assumed to be a consequence or “output”. The goal is to identify other elements in the MIM that could be caused and act as enriched terms. We refer to these elements as identified targets because they are likely to trigger (or counter) the observed changes between samples and thus could be the primary driver of disease pathologies. In contrast to downstream enrichment, the weighting factors are the influence scores from targets to DCEs. The definition of a target depends on the context and nature of the data but is generally not limited to a specific molecule type. For example, targets can refer to elements associated with changes in the expression profiles of genes in a transcriptomics experiment, changes in the concentrations of metabolites in a metabolomics experiment, or changes in the levels of phenotypes. In addition, targets can either be positive, affecting DCEs according to their FC values or negative, having the exact opposite effect. Both may be of interest to the user, as suppression of positive targets or activation of negative targets (or vice versa) serves the same purpose. We rank upstream targets according to their sensitivity (= true positive rate, i.e., ability to affect DCEs) and specificity (= true negative rate, i.e., ability not to affect non-DCEs). Sensitivity is greater than zero for positive targets and less than zero for negative targets. Sensitivity (= true positive rate, Eq. 8)) will be 1 (= positive target) if the influence of *v* on every DCE is 1. For example, a predicted target with a sensitivity of 1 in a transcriptomics experiment refers to a transcription factor that directly induces the expression of all DEGs with a positive FC value and represses the expression of all DEGs with a negative FC value. Conversely, the sensitivity will be −1 (= negative target) if the influence of *v* on every DCE is −1. Specificity (= true negative rate, Eq. 9)) will be 1 if the influence of *v* on every non-DCE is 0.

8 $$\begin{array}{*{20}{c}} {{{{\mathrm{Sensitivity}}}}_v = \frac{{\mathop {\sum }\nolimits_{u \,\in\, {{{\mathrm{DCEs}}}}} (I_{v,u} \cdot {{{\mathrm{FC}}}}_u)}}{{\mathop {\sum }\nolimits_{u \,\in\, {{{\mathrm{DCEs}}}}} (|{{{\mathrm{FC}}}}_u|)}}} \end{array}$$

9 $$\begin{array}{*{20}{c}} {{{{\mathrm{Specificity}}}}_v = \frac{{\mathop {\sum }\nolimits_{u \,\notin\, {{{\mathrm{DCEs}}}}} (1 - |I_{v,u}|)}}{{\mathop {\sum }\nolimits_{u \,\notin\, {{{\mathrm{DCEs}}}}} (1)}}} \end{array}$$

Statistics for upstream enrichment are performed similarly to the downstream enrichment, however, using upstream influence values instead. When identifying upstream targets, ES should also depend on DCEs that are not included in the element set of the enriched target. Therefore, unlike downstream enrichment, we include FC values of DCEs that are not regulated by *v* as the parameter *k* from Eq. 6, resulting in the adapted ES (Eq. 10)).

10 $${{{{\mathrm{ES}}}}_v = \frac{{\mathop {\sum }\nolimits_{u \in {{{\mathrm{DCEs}}}}} (|I_{u,v} \cdot {{{\mathrm{FC}}}}_u| \cdot I_{u,v} \cdot {{{\mathrm{FC}}}}_u)}}{{{\mathop {\sum }\nolimits_{{u \in {\mathrm{DCEs}}}}\atop {{{\;\;} I_{u,v} = 0}}} ({{{\mathrm{FC}}}}_u) + \mathop {\sum }\nolimits_{u \in {{{\mathrm{DCEs}}}}} (I_{u,v}^2 \cdot {{{\mathrm{FC}}}}_u^2)}}}$$

### Implementation as a MINERVA plugin

We developed a JavaScript-based plugin suite for the MINERVA platform, which implements our 2DEA approach in an intuitive user interface. The plugin suite is loaded into MINERVA through the main plugin file from GitHub, which then loads an additional file for each plugin (Xplore.js and Omics.js) as well as additional JavaScript and CSS files. The underlying annotated MIM data is fetched from the same directory as two separate JSON files (Elements.json and Interactions.json) for nodes and edges of the network, respectively. The plugins can be adapted for other disease map projects, given that corresponding data files are generated, which is described in more detail on the AIR website: https://air.bio.informatik.uni-rostock.de/plugins.

For the case studies in this manuscript, we implement the plugins using the MIM of the AIR, whose curation has been described previously^11^. The complete AIR MIM contains more than 6500 elements connected by a total of over 22,000 interactions. Of the latter, approximately 12,000 are positive and 9800 are negative. The elements include more than 90 phenotypes, 250 metabolites, 4700 proteins, 290 complexes, 460 miRNAs, and 410 lncRNAs.

The plugin code and data files are available at: https://github.com/sbi-rostock/AIR/tree/master/AirPlugins.

### Case study input preparation

For the case studies, we analyzed murine colitis RNA-seq data, for which we downloaded raw read counts from GEO (Accession number GSE131032). The data were analyzed using the R DESeq2 package, comparing each of the eight samples from day 2 onwards with the day zero control. For comparing 2DEA with GSEA, we selected DEGs (adj. *p* value < 0.05 by DESeq2) from each of the eight time points as the DCE input list for both approaches. Gene sets for GSEA were created for each phenotype using all elements from submaps in the AIR with an influence score other than zero. For IFNα stimulated B-cells, we directly used GEO’s GEO2R^36^ to compute FC values and adjusted *p* value for four samples vs. the control (no IFNα) (Accession number GSE75194). For each dataset, we summarized their results in a tab-separated text file containing the gene name in the first column together with the FC and adj. *p* value for each comparison, respectively, as additional columns. These files were uploaded to the plugins for further analysis.

### Reagents and tools table

**Table:** 

Resource/ Software	Reference or Source
**Plugin Development**	
npm v6.14.4	https://www.npmjs.com/
Chart.js v3.5.1	https://www.chartjs.org/
Decimal.js v10.3.1	https://mikemcl.github.io/decimal.js/
**Data analysis**	
RStudio v1.4.1106	http://www.rstudio.com/
DESeq2 for R v1.28.1	Love et al.^37^
**SBML visualization**	
CellDesigner v4.4.2	Funahashi et al.^10^
**GSEA**	
GSEAPy v0.10.5	https://gseapy.readthedocs.io/

## Supplementary information

Suppplementary File

## Data Availability

Data files are available from GitHub at: https://github.com/sbi-rostock/AIR/tree/master/AirPlugins. Case Study datasets were fetched from NCBI Gene Expression Omnibus (GEO) with the accession numbers GSE131032 and GSE75194.

## Code availability

JavaScript files of the plugins are available from GitHub at: https://github.com/sbi-rostock/AIR/tree/master/AirPlugins.

## References

[1] Mazein A (2018). Systems medicine disease maps: community-driven comprehensive representation of disease mechanisms. npj Syst. Biol. Appl..

[2] Ostaszewski M (2019). Community-driven roadmap for integrated disease maps. Brief. Bioinform.

[3] Fujita KA (2014). Integrating pathways of Parkinson’s disease in a molecular interaction map. Mol. Neurobiol..

[4] Singh V (2018). Computational systems biology approach for the study of rheumatoid arthritis: from a molecular map to a dynamical model. Genomics Comput. Biol.

[5] Mazein A (2018). AsthmaMap: an expert‐driven computational representation of disease mechanisms. Clin. Exp. Allergy.

[6] Parton A, McGilligan V, Chemaly M, O’Kane M, Watterson S (2019). New models of atherosclerosis and multi-drug therapeutic interventions. Bioinformatics.

[7] Ostaszewski M (2021). COVID19 disease map, a computational knowledge repository of virus–host interaction mechanisms. Mol. Syst. Biol..

[8] Keating SM (2020). <scp>SBML</scp> Level 3: an extensible format for the exchange and reuse of biological models.. Mol. Syst. Biol..

[9] Kitano H, Funahashi A, Matsuoka Y, Oda K (2005). Using process diagrams for the graphical representation of biological networks. Nat. Biotechnol..

[10] Funahashi, A., Morohashi, M., Matsuoka, Y., Jouraku, A. & Kitano, H. in Choi, S. (eds) *Introduction to Systems Biology.* Ch. 21 (Humana Press, 2007).

[11] Serhan CN (2020). The atlas of inflammation resolution (AIR). Mol. Aspects Med..

[12] Gawron P (2016). MINERVA—a platform for visualization and curation of molecular interaction networks. npj Syst. Biol. Appl..

[13] Jiao X (2012). DAVID-WS: a stateful web service to facilitate gene/protein list analysis. Bioinformatics.

[14] Bindea G (2009). ClueGO: a cytoscape plug-in to decipher functionally grouped gene ontology and pathway annotation networks. Bioinformatics.

[15] Chen EY (2013). Enrichr: interactive and collaborative HTML5 gene list enrichment analysis tool. BMC Bioinformatics.

[16] Subramanian A (2005). Gene set enrichment analysis: a knowledge-based approach for interpreting genome-wide expression profiles. Proc. Natl Acad. Sci. USA.

[17] Köhler S (2021). The human phenotype ontology in 2021. Nucleic Acids Res..

[18] Kanehisa M (2000). KEGG: Kyoto encyclopedia of genes and genomes. Nucleic Acids Res..

[19] Kanehisa M, Furumichi M, Tanabe M, Sato Y, Morishima K (2017). KEGG: new perspectives on genomes, pathways, diseases and drugs. Nucleic Acids Res..

[20] Martens M (2021). WikiPathways: connecting communities. Nucleic Acids Res..

[21] Gerstner N (2020). GeneTrail 3: advanced high-throughput enrichment analysis. Nucleic Acids Res..

[22] Hong G, Zhang W, Li H, Shen X, Guo Z (2014). Separate enrichment analysis of pathways for up- and downregulated genes. J. R. Soc. Interface.

[23] Warden CD, Kanaya N, Chen S, Yuan Y-C (2013). BD-Func: a streamlined algorithm for predicting activation and inhibition of pathways. PeerJ.

[24] Zito A (2021). Gene set enrichment analysis of interaction networks weighted by node centrality. Front. Genet..

[25] Catlett, N. L. et al. Reverse causal reasoning: applying qualitative causal knowledge to the interpretation of high-throughput data. *BMC Bioinformatics* **14**, 340 (2013). 10.1186/1471-2105-14-340 PMC4222496 24266983

[26] Krämer A, Green J, Pollard J, Tugendreich S (2014). Causal analysis approaches in ingenuity pathway analysis. Bioinformatics.

[27] Hoksza D, Gawron P, Ostaszewski M, Smula E, Schneider R (2019). MINERVA API and plugins: opening molecular network analysis and visualization to the community. Bioinformatics.

[28] Czarnewski P (2019). Conserved transcriptomic profile between mouse and human colitis allows unsupervised patient stratification. Nat. Commun..

[29] Mostafavi S (2016). Parsing the interferon transcriptional network and its disease associations. Cell.

[30] Ivashkiv LB, Donlin LT (2014). Regulation of type I interferon responses. Nat. Rev. Immunol..

[31] Gal-Ben-Ari S, Barrera I, Ehrlich M, Rosenblum K (2019). PKR: a kinase to remember. Front. Mol. Neurosci..

[32] Chee, A. V., Lopez, P., Pandolfi, P. P. & Roizman, B. Promyelocytic leukemia protein mediates interferon-based anti-herpes simplex virus 1 effects. *J. Virol*. **77**, 7101–7105 (2003). 10.1128/JVI.77.12.7101-7105.2003 PMC156157 12768029

[33] Lu, R., Au, W. C., Yeow, W. S., Hageman, N. & Pitha, P.M. Regulation of the promoter activity of interferon regulatory factor-7 gene. Activation by interferon snd silencing by hypermethylation. *J. Biol. Chem*. **275**, 31805–31812 (2000). 10.1074/jbc.M005288200 10924517

[34] Benjamini Y, Hochberg Y (1995). Controlling the false discovery rate: a practical and powerful approach to multiple testing. J. R. Stat. Soc. Ser. B.

[35] van Iterson M, Boer JM, Menezes RX (2010). Filtering, FDR and power. BMC Bioinformatics.

[36] Sean D, Meltzer PS (2007). GEOquery: a bridge between the gene expression omnibus (GEO) and BioConductor. Bioinformatics.

[37] Love MI, Huber W, Anders S (2014). Moderated estimation of fold change and dispersion for RNA-seq data with DESeq2. Genome Biol.

[38] Boyle EI (2004). GO::TermFinder—open source software for accessing gene ontology information and finding significantly enriched gene ontology terms associated with a list of genes. Bioinformatics.

[39] Martignetti L, Calzone L, Bonnet E, Barillot E, Zinovyev A (2016). ROMA: representation and quantification of module activity from target expression data. Front. Genet..

[40] Tarca AL, Draghici S, Bhatti G, Romero R (2012). Down-weighting overlapping genes improves gene set analysis. BMC Bioinformatics.

