# Supplementary material for: Network- and enrichment-based inference of phenotypes and targets from large-scale disease maps
Source: NPJ Syst Biol Appl. 2022 Apr 26;8:13. doi: 10.1038/s41540-022-00222-z (PMC9042890; doi:10.1038/s41540-022-00222-z)
Supplement: Supplementary file 1 — Suppplementary File [file 41540_2022_222_MOESM1_ESM.pdf]

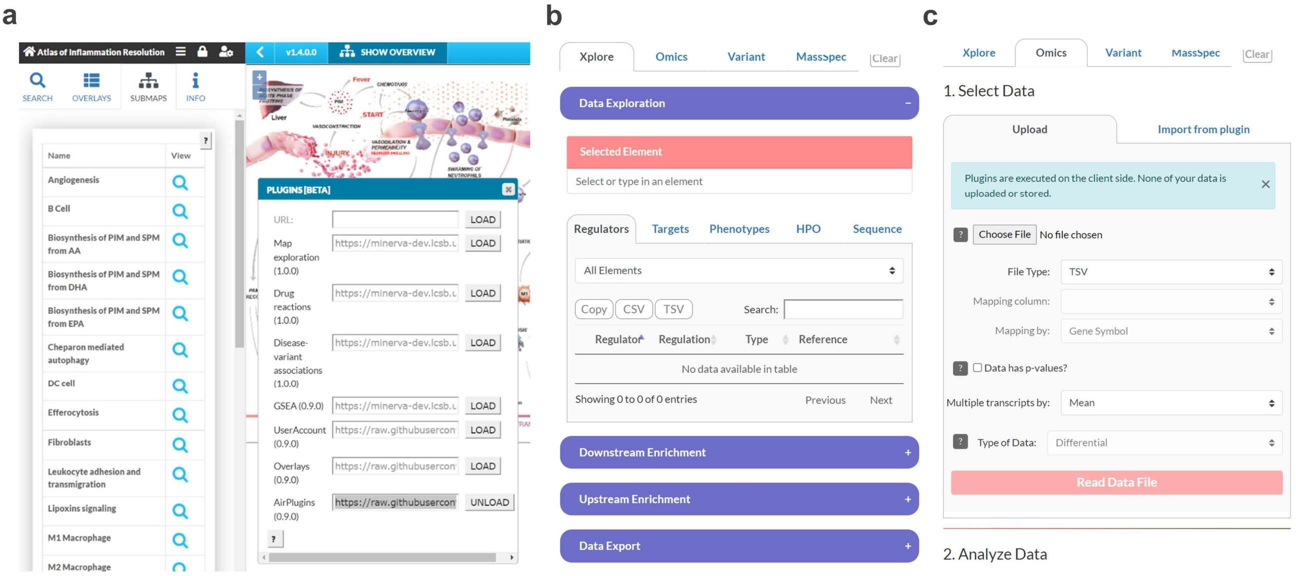

Supplementary Figure 1: User Interface (UI) of the AirPlugins in MINERVA. Screenshots of how to initialize the plugins on the AIR (A) and the initial UIs of the Xplore (B) and Omics (C) plugins.

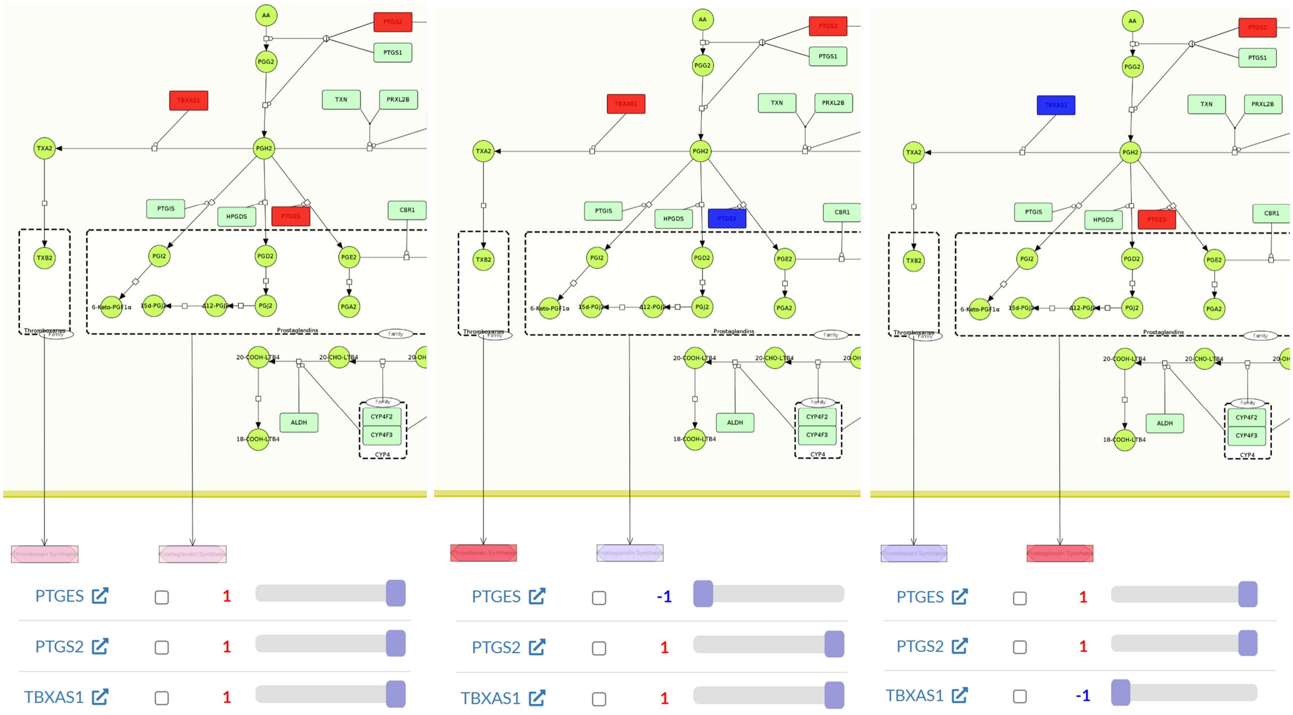

Supplementary Figure 2: *In silico* perturbations with the Xplore Plugin on the submap "Biosynthesis of SPM and PIM from arachidonic acid" in the Atlas of Inflammation Resolution. An intuitive GUI allows users to perturb selected elements by setting a custom log2 fold change value between -1 and 1. Setting the value automatically highlights the respective SBGN elements in the submaps with the perturbed value and the predicted impact on downstream phenotypes.

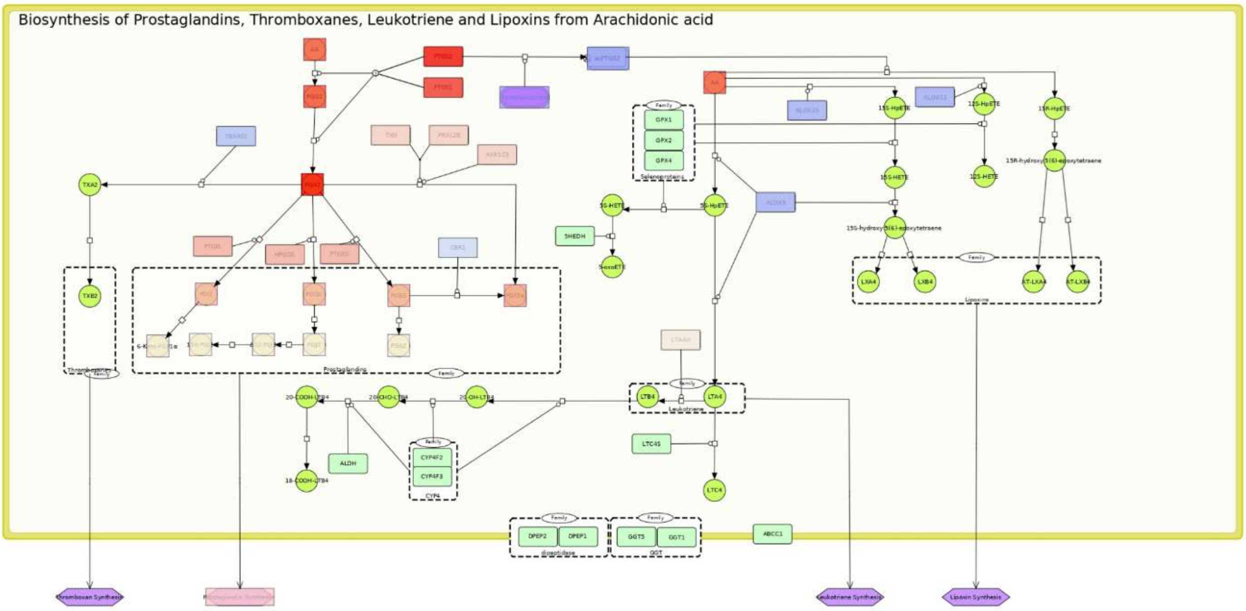

Supplementary Figure 3: The Biosynthesis of PIM and SPM from AA submap with highlighted phenotype influence values for all elements regulating prostaglandin synthesis (bottom). Red: positive regulators, Blue: negative regulators. Hub metabolites, e.g., PGH2, and key enzymes, e.g., cyclooxygenase 2 (PTGS2), are the highest influencing elements. Enzymes that metabolize elements from the pathway have a negative influence.
